# Wave-like behaviour in (0,1) binary sequences

**DOI:** 10.1038/s41598-022-18360-z

**Published:** 2022-08-17

**Authors:** Enrique Canessa

**Affiliations:** The Abdus Salam International Centre for Theoretical Physics, ICTP, Trieste, 34151 Italy

**Keywords:** Genome, Structural variation, Biological physics, Bioinformatics

## Abstract

A comprehensive study of the properties of finite (0,1) binary systems from the mathematical viewpoint of quantum theory is presented. This is a quantum-inspired extension of the GenomeBits model to characterize observed genome sequences, where a complex wavefunction $\psi _{n}$ is considered as an analogous probability measure and it is related to an alternating (0,1) binary series having independent distributed terms. The real and imaginary spectrum of $\psi _{n}$ *vs.* the nucleotide base positions display characteristic features of sound waves. This approach represents a novel perspective for identifying and “observing” emergent properties of genome sequences in the form of wavefunctions via superposition states. The motivation is to develop a simple algorithm to perform wave calculations from binary sequences and to apply these wave functions to sonification.

## Introduction

The derivation of analogous relationships between different disciplines has become increasingly popular in recent years. This type of approach has been implemented in a wide range of applications, as for example in the domains of physics and finance. These efforts have pursued analogies between stochastic models commonly used in the statistical physics of complex systems and stock market dynamics^1^. The thermodynamic interpretation of multifractality was established in^2^, and an expression for an analogous specific heat in these systems was derived in^1^.

Another particularly interesting example is the 3D deformation of a compressible filament which has been modelled as the oscillation of a relativistic non-linear pendulum, where the compression modulus relates to the relativistic particle’s rest mass, and the bending modulus mimics the speed of light^3^. A mathematical approach for the quantum information representation of biosystems has been introduced in^4^. In this study, biological functions such as psychological functions and epigenetic mutation are modelled in analogy with the physics of open quantum systems. In^5^, it has been argued how analogous Hawking radiation-like phenomena may arise in chaotic systems with exponential sensitivity to initial conditions (butterfly effect). There exists also suggestive parallels between various aspects of number theory and physics phenomena^6^.

Such parallel analyses have been useful to describe the rich complexity of diverse dynamical systems in terms of well-known physics phenomena and, therefore, best characterize the peculiarities of their behaviour. Motivated by these analogous representations, we apply in this work quantum formalism outside of physics to derive properties of binary sequences containing 0 and 1 distinct outcomes from a new perspective.

The purpose of this work is to introduce a wave-like function for complete genome sequences of pathogens represented by an alternating binary series having independently distributed terms associated with (0,1) binary indicators for the nucleotide bases. This quantum-based mathematical description is an extension of our previous GenomeBits model^7,8^. It can reveal further unique imprints of the genome dynamics at the level of nucleotide ordering for different systems (or genome mutations) following experimental measures over *N* intervals. This novel approach allows to identify and ”observe” emergent properties of genome sequences in the form of wavefunctions via superposition states from a new perspective. We also compare results with random binary sequences.

Following Ref.^4^, the present approach is treated from the viewpoint of quantum theory as a measurement theory and not as being the atomic-level modelling of real quantum physical processes. The complex wavefunction is seen as a mathematical description of an isolated, analogous quantum system. The real and imaginary parts of the longitudinal wavefunction *vs.* the nucleotide bases display analogous features of sound waves. The examples given in this context are selected as test for the mathematical conversion of binary sequences into isolate acoustic-like waves by appropriate formulas. Our motivation is to develop a simple algorithm to perform wave calculations from binary sequences and to apply these wave functions to sonification where inputs of zeros and ones are sufficient. The use of non-speech sounds in the biological context can be useful to identify trends in gene sequences and determinate its properties. For example, sonification algorithms based on biological rules for DNA sequence data use codons to generate strings of audio that are representative of ribonucleotides synthesized during transcription (see^9^).

## Binary sequences formulae

Let us consider the quantitative GenomeBits method for the examination of distinctive patterns of complete genome data^7,8^. It consists of a certain type of finite alternating series having terms converted to (0,1) binary values for the nucleotide bases $\alpha = A,C,T,G$ as observed along the reported genome sequences, with (A)denine, (C)ytosine, (G)uanine and (T)hymine –or (U)racil RNA genome for single strand. In other words, the GenomeBits approach defines the sum relation

1 $$\begin{aligned} \Phi (X_{\alpha ,k}) = \phi (X_{\alpha ,1}) + \cdots + \phi (X_{\alpha ,k}) = \sum _{j=1}^k \phi (X_{\alpha ,j})= \sum _{j=1}^k (-1)^{j-1}X_{\alpha ,j} \; , \end{aligned}$$

where the individual values $X_{j} = 0$ or $X_{j} = 1$ are associated according to their position *k* along the genome sequences of length *N*, satisfying the following relation

2 $$\begin{aligned} X_{\alpha ,k} = | \Phi (X_{\alpha ,k}) - \Phi (X_{\alpha ,k-1}) | \; . \end{aligned}$$

The arithmetic progression carries positive and negative signs $(-1)^{j-1}$ and a finite non-zero first moment of the independently distributed variables $X_{\alpha ,j}$. By default, plus and minus signs are chosen sequentially starting with $+1$ at $j=1$.

The mapping of Eq. (1) into four binary projections of the $\alpha$-sequences follows the three-base periodicity characteristic of protein-coding DNA sequences studies in^10^. Analysing genomics sequencing via this class of finite alternating sums allows to extract unique features at each base. From the view of statistics, such series are equivalent to a discrete-valued time series for the statistical characterization of random data sets^1^. In the following, however, the above GenomeBits relation is to be considered as a resulting wave created by a certain superposition of (one or more discretized) wavefunctions, *i.e.*, $\phi (X_{\alpha ,N}) \rightleftharpoons \psi _{n}(X_{\alpha ,N})$ in some medium. In general wavefunctions are complex functions and the displacement of this wave is to be a function of base position *k*.

Let us then consider the polar form

3 $$\begin{aligned} \psi _{n}(X_{\alpha ,k})\equiv & {} A\; (-1)^{k-1} | \Phi (X_{\alpha ,k}) - \Phi (X_{\alpha ,k-1}) | \; \exp \left\{ \frac{n\pi i}{\lambda _{_{N}}}\Phi (X_{\alpha ,k})\right\} \; , \nonumber \\= & {} A\; (-1)^{k-1}X_{\alpha ,k} \; \exp \left\{ \frac{n\pi i}{\lambda _{_{N}}} \sum _{j=1}^k (-1)^{j-1}X_{\alpha ,j}\right\} \; , \end{aligned}$$

with *A* a real constant and $n = 1,2 \cdots$

The normalization condition via the complex conjugate $\sum _{k=1}^N \psi _{n}(X_{\alpha ,k})\psi _{n}^{*}(X_{\alpha ,k}) = \sum _{k=1}^N |\psi _{n}(X_{\alpha ,k})|^{2} = 1$ implies

4 $$\begin{aligned} A^{2}\sum _{k=1}^N |(-1)^{k-1}X_{\alpha ,k}|^{2} = A^{2}\sum _{k=1}^N X_{\alpha ,k}^{2} = 1 \; . \end{aligned}$$

Since $X_{\alpha ,k}$ may take (0,1) values only, one then gets the amplitude

5 $$\begin{aligned} A = \frac{1}{\pm \sqrt{N_{_{+1}}}} \; , \end{aligned}$$

where $N_{_{+1}}$ is the total number of 1’s found in the complete $N = N_{_{0}} + N_{_{+1}}$ sequences for each species $\alpha$.

To simplify calculations, let us choose

6 $$\begin{aligned} \lambda _{_{N}} \equiv \sum _{j=1}^N \phi (X_{\alpha ,j}) = \sum _{j=1}^N (-1)^{j-1}X_{\alpha ,j} = \Phi (X_{\alpha ,N})\; , \end{aligned}$$

which corresponds to the maximum real value for the alternating sum of binary sequences. From Eq. (3) and Euler’s identity, it is interesting to note that for $k \rightarrow N$ the Cartesian form

7 $$\begin{aligned} \psi _{n}(X_{\alpha ,N}) = \left( \frac{1}{\pm \sqrt{N_{_{+1}}}}\right) (-1)^{N-1} X_{\alpha ,N} \; [\cos (n\pi ) + i \;\sin (n\pi )] = \frac{(-1)^{n}}{\pm \sqrt{N_{_{+1}}}}\; \phi (X_{\alpha ,N}) \; . \end{aligned}$$

oscillates and decreases for large numbers of 1’s and $\forall n \ge 1$. Hence, the GenomeBits Eq. (1) for complete genome sequences can be seen as a resulting wave in steady state created by certain non-zero complex wavefunctions $\psi$ that change with $k \ne 0$ and have same maximum density probability $1/N_{_{+1}}$ at each *n*.

## Results and discussion

**Figure 1:**
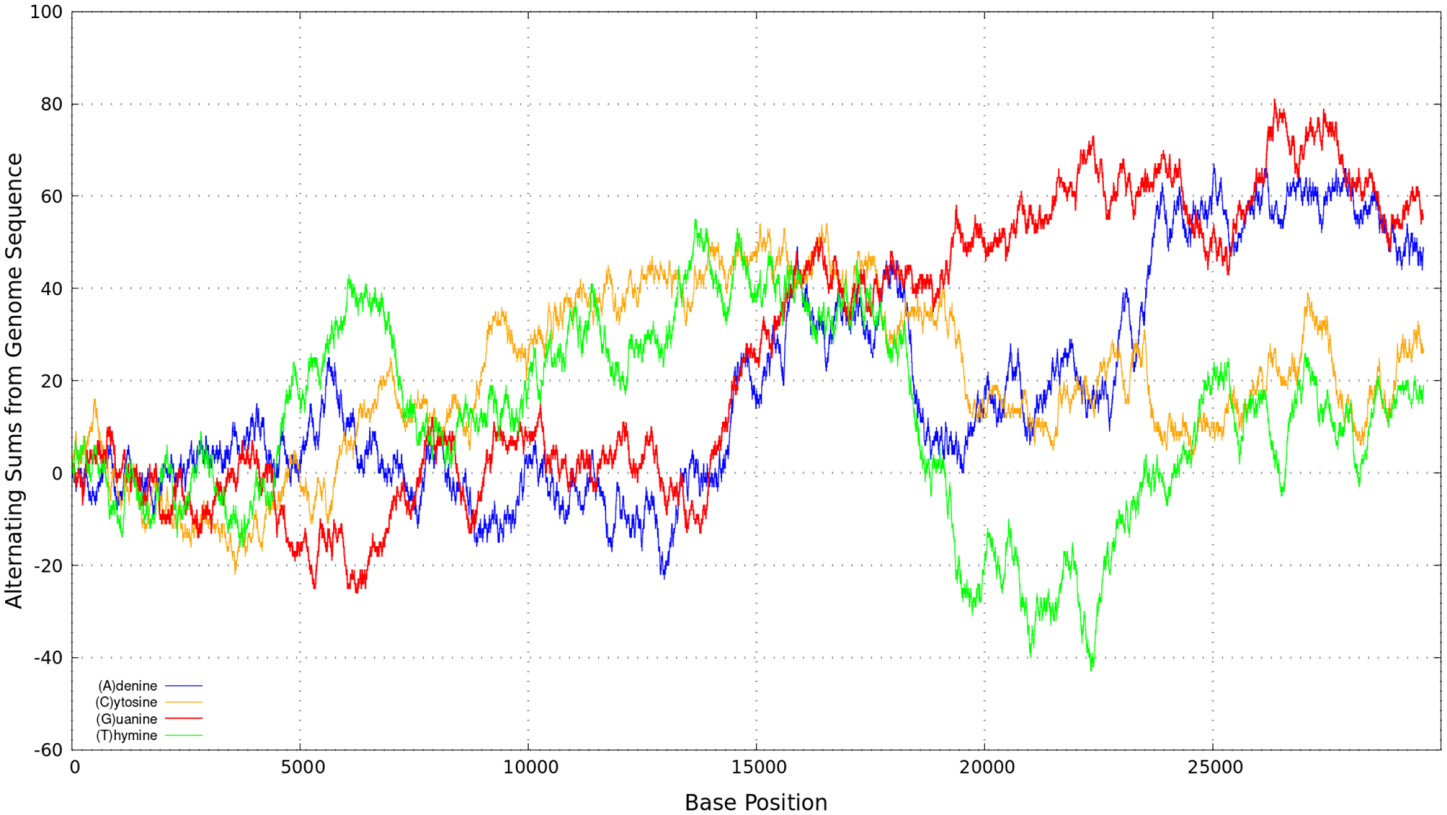
Binary sequence sum series of Eq. (1) for representative nucleotide bases from the genome sequence of coronavirus (Omicron variant, GISAID EPI_ISL_11901306^11^).

In this section we discuss a case study addressed to show the applicability of the above formulae. The examples include cases of wavefunctions with random (0,1) sequences with either different densities of random 0’s and 1’s –namely, 70% number of zeros and 30% of ones, as well as a wavefunction for a representative full-length GenomeBits sequence of coronavirus (Omicron variant)^7,8^. We select the new variant of concern Omicron Lineage B.1.1.529 since this is the variant of coronavirus most dominant and more infectious around the world at the time of this writing (with almost 1.2 million cases). It emerged with more than a dozen spike mutations from southern Africa in Nov.2021. For comparison, we shall also consider in this study the original coronavirus Wuhan-Hu-1 of China. Illustrative results derived for the sums in Eq. (1) for nucleotides of the Omicron strand of complete genome sequence are shown in Fig. 1. See Ref.^7^ for the GenomeBits analysis of Eq. (1) for the first pathogen variants arisen over the course of the pandemic since early 2020.

The GISAID accession number for the full-length genome sequencing shown in Fig. 1 is EPI_ISL_11901306 dated 2022-04-04 from Monza, Italy^11^, with $N=29613$ base pairs. It is interesting to note that for $\alpha = (A)$ the total numbers of zeros is 20770 (70%) and the total number of ones is 8843 (30%). For $\alpha = (C)$ these correspond to 24199 (82%) and 5414 (18%), respectively. For $\alpha = (G)$ one finds 23809 (80%) and 5804 (20%) and, similarly, for $\alpha = (T)$ the values become 20061 (68%) and 9552 (32%). There are regions in this figure where these binary projections reveal some distinctive oscillatory patterns and interesting unique imprints of the intrinsic gene organization at the level of nucleotides.

Remarkably, the signals in Fig. 1 are essentially similar in behaviour. The positive and negative terms in the sums of the discrete (0,1) values partly cancel out, allowing the series to diverge from zero rapidly and to become a non-Cauchy sequence type. As shown next, these statistical representations are powerful to targeting the proposed complex-valued wavefunctions of Eq. (3).

**Figure 2:**
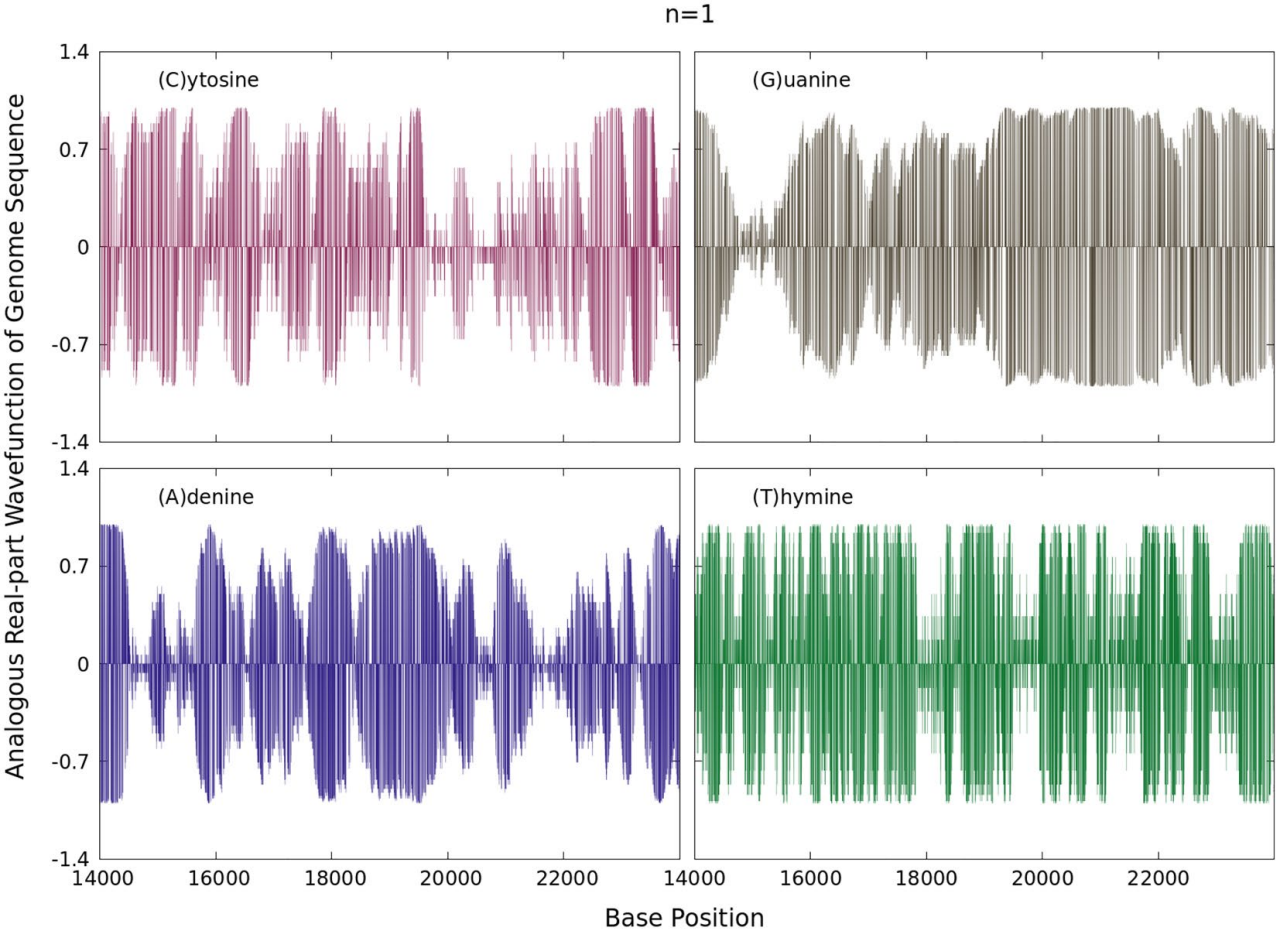
Real parts of the wavefunctions in Eq. (3) for $n=1$ for each sum series displayed in Fig. 1.

**Figure 3:**
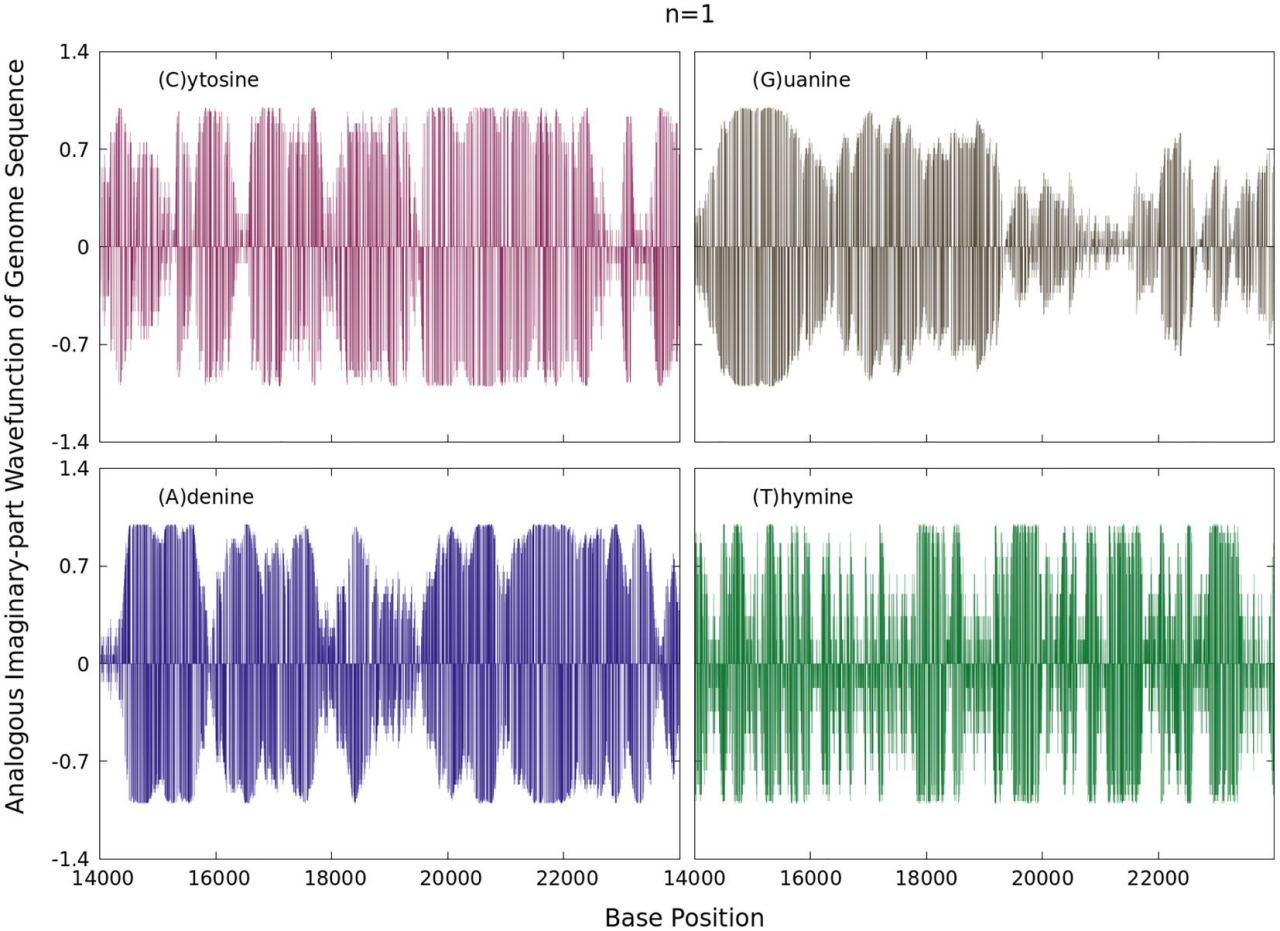
Imaginary parts of the wavefunctions in Eq. (3) for $n=1$ for each sum series displayed in Fig. 1.

The corresponding real and imaginary parts of $\psi _{1}(X_{\alpha ,k})$ at a first analogous bound state are depicted in Figs. 2 and 3 for the sum series of Fig. 1 along the base positions $14000 \le k \le 24000$ and normalized by *A*. This wavefunction is here understood as the mathematical description of an analogous ground state of an isolated quantum system. The real and imaginary parts may in turn resemble analogous oscillatory acoustic waves in all sequences considered. Such sequences share zero values in $\mathbb {R}$ when $X_{\alpha ,k}=0$. Individually all the wavefunctions for a given *n* exhibit a set of zeros in their real and imaginary parts along the complete real $1 \le k \le N$ axis.

These results are a consequence of factorizing the full wavefunction as the product of two different expressions *i.e.*, a linear function proportional to the (0,1) binary sequences and a complex exponential form. Since this wavefunction is discretized, one may think of it as a standing wave with zero nodes. From the numerical point of view, these wavefunctions are here not quantum states of interest with a definite total physics energy, but rather an analogous mathematical construction. At any other point different from zero, the wavefunction with oscillatory behaviour for genome sequences is obtained. This is a remarkable result based on a simple algorithm of finite alternating sum series having independently distributed terms associated with just two indicators (0,1) for each of the nucleotide bases *A*, *C*, *T* or *G*. In this light, it is worthy to note that previous algorithmic conversions for protein sequences and multiple sequence alignments to oscillatory sound waves has been reported in^12^, in which each amino acid (or in the case of Algorithm IV –a summary of variation in amino acids at a position in a multiple sequence alignment) is represented by a specific musical note. Algorithmic conversion of DNA sequence data to sound has been reported in^13^.

**Figure 4:**
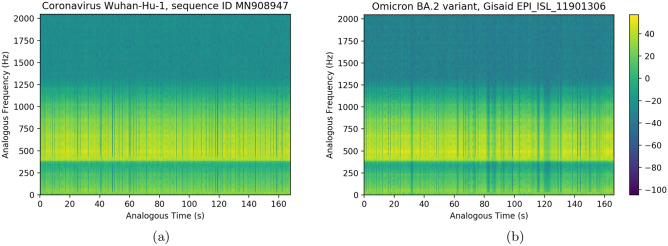
Examples of analogous time-frequency representation of the strand of nucleotide bases (A)denine produced from a wav audio file generated via the GenomeBits wavefunction for (**a**) the original coronavirus Wuhan-Hu-1 of China (ID MN908947.3) and (**b**) the GenomeBits wavefunction for Omicron subvariant BA.2 in Fig. 1. The latter can be downloaded from GitHub^14^. A visual comparison could help to identify virus mutations.

The wav audio files generated via the present extended GenomeBits wavefunctions, by transforming the occurrence of nucleotides of the same type along the genome sequences can be downloaded from GitHub^14^. An analogous time-frequency representation of the strand of genome data, showing homogeneous peak signals over time, is shown in Fig. 4 in the form of spectrogram. Each data point in the audio curves has been fitted to a gaussian function to correlate a continuous audio spectrum in the analysis of our waveform reconstruction. In these calculations, we use similar parameters as in^15^: sample rate: 4096, precision: 16-bit, duration: 2:46.28 min for 681097 samples: file size: 1.36M: bit rate: 65.5k and sample encoding: 16-bit in 1 channel. This is a visual method that could help to identify and quantify (future) virus mutations. For example, in Fig. 4 the spectrograms obtained from an audio file generated via the GenomeBits wavefunction for the complete genome sequences of the original coronavirus Wuhan-Hu-1 of China (ID MN908947.3) and for the Omicron subvariant BA.2 plotted in Fig. 1 (EPI_ISL_11901306) can be compared by visual inspection. As done in^15^, we have artificially shifted these curves in frequency by an offset of 400Hz to have a more clear picture when assessing significant variations through, *e.g.,* the different densities of blue lines. One can see how the frequencies derived from each sequence vary over time on the spectrogram, which correspond to how the audio actually sounds. In passing, we mention that gravitational waves from a binary black hole merger have been recently sonificated in^15^, and music from fractal noise have been also investigated in^16^.

The present novel method of binary projections introduced here has drawn inspiration from quantum theory. The main point is to uncover distinctive patterns out of some intrinsic organization embedded in the sequences, including acoustic-like waves. Our work differs from other formalism in that sequence variations are characterized by simply associating (0,1) indicators for each nucleotide bases (A), (C), (G) and (T) separately.

**Figure 5:**
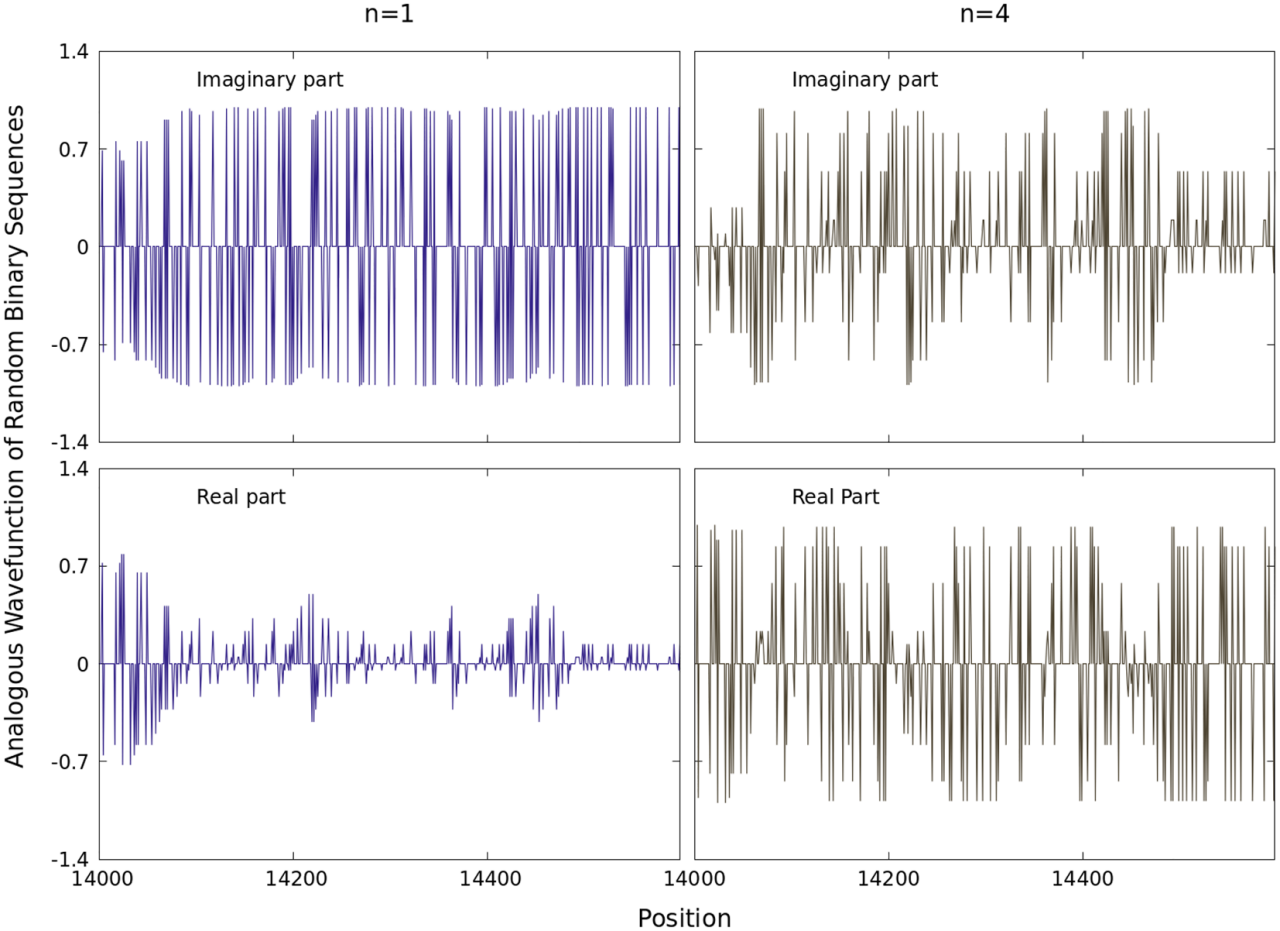
Real and Imaginary parts of the wavefunctions in Eq. (3) for binary random numbers with different frequencies of 0’s (70%) and 1’s (30%) and two different *n*’s.

Inspired by the above findings in the genome sequences of coronavirus, we include in Fig. 5 examples addressed to show that, in general, there are common patterns in the behaviour around the zeros of the real and complex parts of the wavefunction $\psi _{1}(X_{\alpha ,k})$ for random (0,1) sequences with 70% of number of zeros and 30% of ones. For different sequences randomly generated in arbitrary form and for two different values of *n*, it follows that the maxima and minima in the curves differ in shape. For $n=1$ the profile of the imaginary part of $\psi$ presents an analogous ”white noise” type of behaviour, which carries the least correlations. As for Fig. 3, the occurrence of a particular bit is essentially independent of previous or future values of the binary expansion^17^.

**Figure 6:**
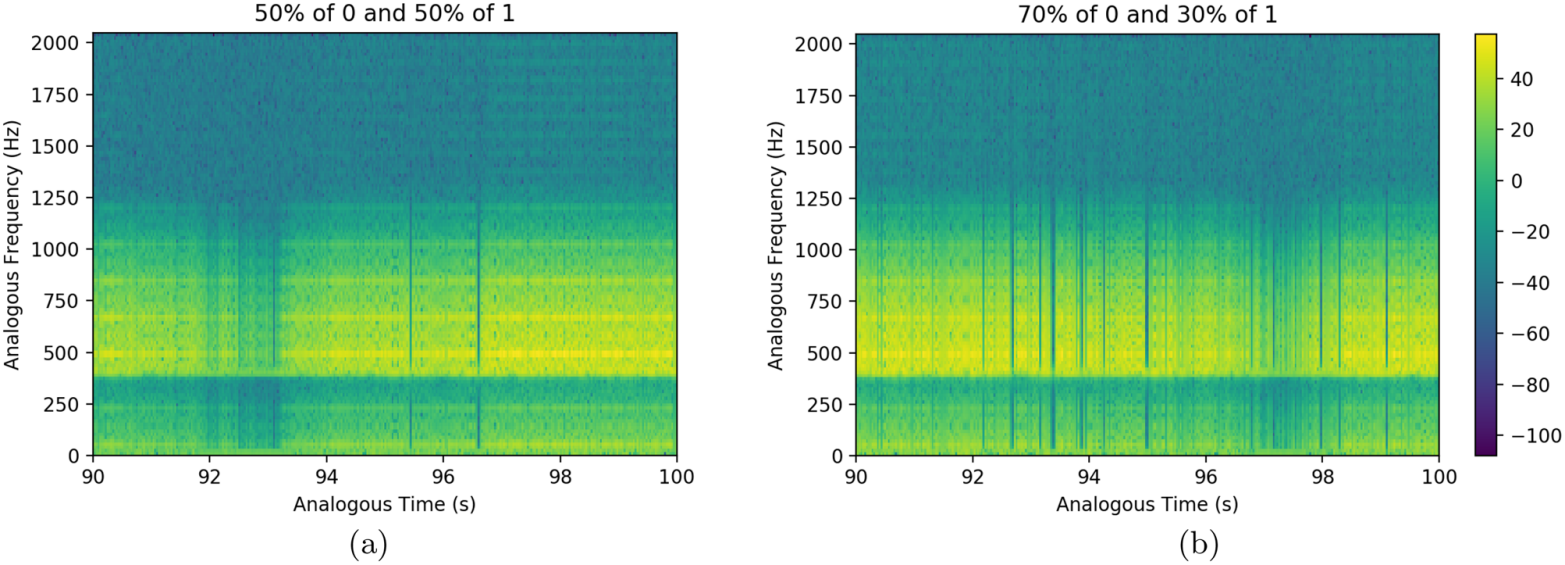
Examples of ”music” from random sequences.

To a first glance, the ”music” of the random sequences in Fig. 6 (for $n=1, \cdots$) may look similar to that obtained by the sequences of the real virus (Fig. 2 and 3). However, the present model grasp some information through the densities of the blue lines which appear to be higher at some analogous time intervals. This can be perceived by inspection of Fig. 6 for two different random sequences with (50% of 0, 50% of 1) and (70% of 0, 30% of 1), and $n=1$.

Closing this discussion, it seems sensible to interpret our quantum inspired Eq. (3) as an inherent mathematical method to study oscillations in binary systems although its nature may appear abstract. We stress that classical mathematics still plays a crucial role to describe the rich complexity of biological systems and peculiarities of their behaviour^4^.

## Summary

Our previous GenomeBits method has been here extended to present a novel perspective for identifying and ”observing” emergent properties of DNA sequences in the form of wave functions^7,8^. Starting with an extension of the alternating sums of genome sequences as in Eq. (1) (which may be seen as a particular case of polynomial associated to some immanant of a matrix for DNA graph^18^), we presented a new quantitative test for comparing natural binary numbers sequences through the complex wavefunction of Eq. (3). Its analogous cumulative probability over all possible base positions is unity. This measure is aimed at finding out a characterization and understanding of sequence variation from a comparative investigation across multiple binary systems including genome sequences and random numbers. The analogous displacement of the wave $X_{\alpha ,k}$ has been chosen satisfying the particular form given by Eq. (3) since the GenomeBits sum relation of Eq. (1) is considered as a resulting wave created by a certain superposition of discretized wavefunctions, *i.e.*, $\phi \rightleftharpoons \psi _{n}$. This is verified by the relation in Eq. (7). Although the present analogy is consistent from a formal mathematical perspective, one aspect that can be criticized is the fact that we cannot make yet some kind of physical association to quantize energy in levels, to derive a system momentum, *etc*. The justification for this is that we are dealing with discretized (0,1) sequences in one dimension.

By construction, the wavefunction Eq. (3) at *n* wears properties that are not different from those of other analogous states $n+1$ as can be deduced through Eq. (7). For finite systems, it makes sense to fix the amplitude *A* by the normalization of an analogous total probability as done in Eq. (5). Under base translation by a spacing $k_{a} > k$, the wavefunction is not symmetric and its modulus satisfies $|\psi _{n}(X_{\alpha ,k+k_{a}})|^{2} = |X_{\alpha ,k+k_{a}}|^{2} \ne |X_{\alpha ,k}|^{2}$. Such behaviour is characteristic of the multi-mode spectrum of aperiodic lattices. Additionally, the observations reported here may share a common quantum-like origin with the method introduced in^4^.

The samples worked out in this paper have been chosen as another test for the mathematical conversion of binary sequences to isolate acoustic-like waves by appropriate formulas. Introducing Eq. (3) as an extension of Eq. (1), we developed a simple method to perform wave calculations where inputs of zeros and ones are sufficient. This opens the possibility to reverse the calculations and derive a characteristic precursory alternating sum from a given recorded audio file as input. We believe this novel aspect of our algorithm could stimulate further investigations toward slicing the sounds of nature.

## Data Availability

All genome sequence data analyzed here (with the GISAID accession numbers indicated in the text) are publicly available at GSIAD https://www.gisaid.org. The datasets (audio waves) generated during the current study are available in the open GitHub repository at https://github.com/canessae/GenomeBits-Waves. The datasets generated and analysed during the current study are also available from the corresponding author upon request.
